# Sirtuin Control of Mitochondrial Dysfunction, Oxidative Stress, and Inflammation in Chagas Disease Models

**DOI:** 10.3389/fcimb.2021.693051

**Published:** 2021-06-09

**Authors:** Xianxiu Wan, Nisha Jain Garg

**Affiliations:** ^1^ Department of Microbiology and Immunology, University of Texas Medical Branch, Galveston, TX, United States; ^2^ Institute for Human Infections and Immunity, University of Texas Medical Branch, Galveston, TX, United States

**Keywords:** peroxisome proliferator-activated receptor gamma coactivator 1, reactive oxygen species, sirtuin, Chagas disease, mitochondrial dysfunction

## Abstract

*Trypanosoma cruzi* is a digenetic parasite that requires triatomines and mammalian host to complete its life cycle. *T. cruzi* replication in mammalian host induces immune-mediated cytotoxic proinflammatory reactions and cellular injuries, which are the common source of reactive oxygen species (ROS) and reactive nitrogen species (RNS) during the acute parasitemic phase. Mitochondrial dysfunction of electron transport chain has been proposed as a major source of superoxide release in the chronic phase of infection, which renders myocardium exposed to sustained oxidative stress and contributes to Chagas disease pathology. Sirtuin 1 (SIRT1) is a class III histone deacetylase that acts as a sensor of redox changes and shapes the mitochondrial metabolism and inflammatory response in the host. In this review, we discuss the molecular mechanisms by which SIRT1 can potentially improve mitochondrial function and control oxidative and inflammatory stress in Chagas disease.

## Introduction

*Trypanosoma cruzi* (*T. cruzi*) infection leads to the development of Chagas disease (CD) that is one of the most frequent causes of heart failure and sudden death in the Americas. There are three stages in Chagas disease: the acute phase, the early chronic phase that is also referred as indeterminate phase, and the late chronic disease phase. Shortly after exposure to the parasite, infected individuals develop acute parasitemia when trypomastigotes and amastigotes can be easily detected by microscopic examination of the blood and/or cerebrospinal fluid. In most of the infected individuals, immune response is sufficiently active to control acute parasitemia within 2 to 4 months post-exposure ([[NoAuthor]]). Infected individuals then appear healthy with almost no clinical symptoms of cardiac involvement and may remain in this phase for decades. However, approximately 1/3 of the infected persons progress to clinically symptomatic, chronic disease phase presented with cardiomyopathy and heart failure (Dias et al., 2016; Rassi et al., 2017). Chronically infected individuals may also develop digestive or neurological disorders.

**Figure 1:**
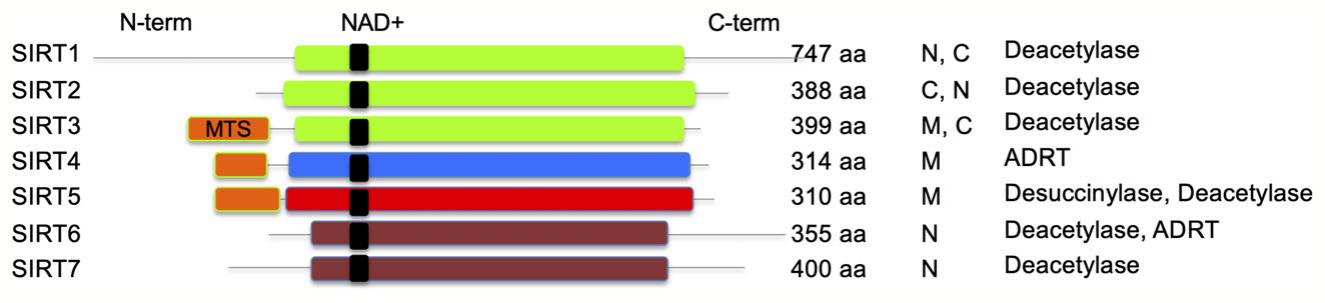
Schematic representation of human sirtuins. SIRT1–3 (class I), SIRT4 (class II), SIRT5 (class III), and SIRT6–7 (class IV) are shown. NAD^+^ binding region is presented in black. MTS, mitochondria-targeting sequence is shown in orange; N, nuclear; C, cytoplasmic; ADRT, ADP-ribosyltransferase. Catalytic histidine and zinc-coordinating cysteines are not shown.

Sirtuins, initially named as silent information regulator 2 (Sir2) proteins, are defined as class III histone deacetylases that utilize nicotinamide adenine dinucleotide (NAD^+^) as substrate. In the deacetylase reaction, sirtuins hydrolyze one NAD^+^ molecule and produce deacetylated substrate, nicotinamide (NAM), and O-acetyl-ADP ribose (Landry et al., 2000). The sirtuin family of proteins in humans consists of SIRT1–SIRT7 that are highly conserved, both structurally and functionally. Sirtuins are ubiquitously expressed in all organs including the blood, brain, heart, kidney, lung, liver, ovary, skeletal muscle, spleen, and testis, though the level of expression varies in different organs. The catalytic core deacetylase domain is highly conserved in sirtuins; however, they differ in sequence, subcellular location, enzyme activity, substrate specificity and physiological functions (Frye, 1999; Michishita et al., 2005) (**Figure 1**). For example, SIRT1, SIRT6, and SIRT7 are generally found in a nuclear compartment and known to modulate gene expression through transcription factors, co-factors, or histones (Liszt et al., 2005; Ford et al., 2006; Mostoslavsky et al., 2006; Michishita et al., 2008; Zhong et al., 2010). SIRT2 is primarily localized in the cytoplasm, and it was shown to regulate oligodendrocyte differentiation and cell cycle (Dryden et al., 2003; Li et al., 2007). The remaining known sirtuins (SIRT3, SIRT4, and SIRT5) are predominantly found in mitochondrial compartment and shown to regulate metabolic enzyme activities and oxidative stress pathways (Ahuja et al., 2007; Nakamura et al., 2008).

SIRT1 is the closest homolog of yeast Sir2, and it is the most studied sirtuin (Frye, 2000). At the cellular level, SIRT1 shuttles between the nucleus and cytoplasm depending on environmental conditions (Hou et al., 2010). SIRT1 has two nuclear localization signals and a coiled-coil domain in addition to the core deacetylase domain. Many of the endogenous substrates of SIRT1, including p53, NBS1, p65, c-Jun, and c-Myc play a role in transcriptional regulation of gene expression (Yeung et al., 2004; Solomon et al., 2006; Yuan et al., 2007; Gao and Ye, 2008; Yuan et al., 2009). While SIRT1 core deacetylase domain determines its enzymatic activity, other domains may influence the binding to NAD^+^ substrate and target proteins. SIRT1-mediated deacetylation activity regulates several proteins and their translation, and it is involved in critical physiological processes including cell proliferation, genomic stability, metabolism, and antioxidant/oxidant response. SIRT1 has been reported to associate with chronic inflammatory diseases, metabolic dysfunctions, neurodegenerative diseases, and cardiovascular dysfunction (Boutant and Canto, 2014; Chang and Guarente, 2014; Herskovits and Guarente, 2014; Hubbard and Sinclair, 2014).

*T. cruzi* is a eukaryotic parasite that consists of genes encoding for two sirtuins, named TcSir2rp1 and TcSir2rp3 that are identified to be localized in cytosol and mitochondria, respectively (Moretti et al., 2015). Inhibition and overexpression studies suggest that TcSir2rp1 and TcSir2rp3 seemingly have opposite roles in parasite growth and differentiation (Moretti et al., 2015). A panel of sirtuin inhibitors has been screened for their activity against TcSir2rp1 and TcSir2rp3 (Matutino Bastos et al., 2020) and broad-range SIRT inhibitors (salermide or nictoinamide) were shown to inhibit *T. cruzi* growth and differentiation in culture and *in vivo* in mice (Moretti et al., 2015). Thus, SIRTs are required for parasite growth and survival in the mammalian host, and SIRT inhibitors are developed as anti-parasite therapies.

In this review, we discuss various roles of SIRT1 with a focus on its beneficial effects in regulating Chagas disease pathogenesis.

## Mitochondria Dysfunction in Experimental Models of CD and Infected Humans

Heart function is supported by a high rate of ATP production through mitochondrial oxidative phosphorylation pathway. Cardiomyocytes have a high copy number of mitochondrial DNA (mtDNA) that encodes essential components of respiratory complexes to support energy demands of the heart (Gustafsson and Gottlieb, 2008). Mitochondrial biogenesis encompasses processes and events resulting in replication, maintenance, and function of mitochondria. Peroxisome proliferator-activated receptor gamma coactivator 1 (PGC-1*α*) is a member of the PGC family of transcription coactivators, and it is designated as a master regulator of mitochondrial biogenesis and oxidative metabolism (Finck and Kelly, 2006). PGC-1*α* is preferentially expressed in the heart, kidneys, and skeletal muscle tissues that have high oxidative capacity and abundant mitochondria. PGC-1*α* functions as an adapter or scaffold protein and docks onto the transcription factor targets or protein complexes to drive the expression of nuclear DNA and mtDNA encoded proteins involved in mitochondrial biogenesis and oxidative phosphorylation (Ventura-Clapier et al., 2008). The estrogen-related nuclear orphan receptors (ERR-*α* and ERR-*γ*) are also activated by PGC-1*α*, which enhances the expression of proteins involved in fatty acid uptake, fatty acid oxidation, and ATP production and transportation (Shao et al., 2010). PGC-1 also binds to and co-activates nuclear respiratory factors 1 and 2 (NRF-1 and NRF-2) that maintain redox homeostasis and upregulate the mitochondrial transcription factor A (TFAM)-mediated mtDNA replication and transcription (Gureev et al., 2019)

**Figure 2:**
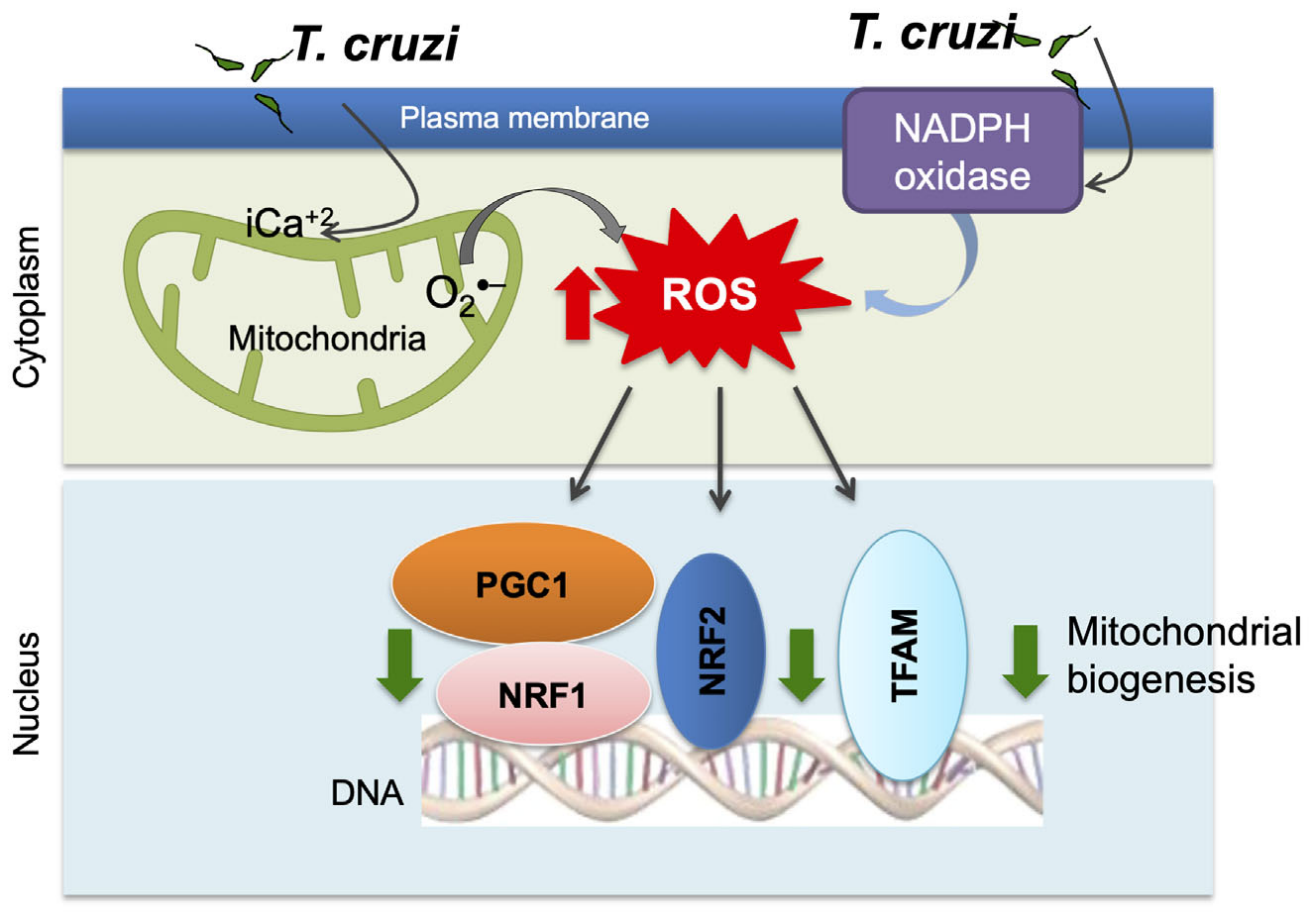
Potential mechanism of mitochondrial dysfunction in Chagas disease. *T. cruzi* uptake by monocytes/macrophages activates NADPH oxidase-mediated superoxide and ROS production. *T. cruzi* induces intracellular Ca^2+^ flux that causes mitochondrial membrane permeability transition, respiratory complex inefficiency, and increased leakage of electron from electron transport chain to oxygen, resulting in superoxide production. ROS suppress mitochondrial biogenesis through inhibition of PGC-1*α*-mediated transcriptional activation of NRF1/2 and TFAM that maintain redox homeostasis and mtDNA replication and transcription.

The impairment of mitochondrial membrane phospholipids, DNA, or proteins can all affect the mitochondrial oxidative phosphorylation capacity. We have documented significant decline in the activities of the respiratory complex I and complex III in cultured human cardiomyocytes and myocardium of mice infected with *T. cruzi (* Vyatkina et al., 2004 *).* Quantitative assays and light and electron microscopic analysis of the human plasma and serum samples (Wen et al., 2006) the myocardial biopsies of human CD patients (Wan et al., 2012) and *T. cruzi-*infected experimental animals showed that mitochondrial degenerative changes occurred in early *T. cruzi* infection and were exacerbated with progression of disease (Wen et al., 2006; Wen et al., 2008; Wan et al., 2012). No changes in PGC-1*α* levels were noted; however, its activity in signaling NRF1/2 and TFAM-mediated mitochondrial biogenesis was clearly compromised as was evidenced by the findings of a decline in cardiac mtDNA content, mtDNA synthesis, and oxidative phosphorylation mediated ATP synthesis in Chagas hearts (Wan et al., 2012) (**Figure 2**).

## Oxidative Stress in CD

High oxidative stress results from increase in the ROS and RNS production above the physiologically relevant threshold levels. ROS describe a variety of free radicals, including superoxide (O_2_•−), hydroxyl radical (•OH), and hydrogen peroxide (H_2_O_2_) derived from molecular oxygen. ROS are formed in various organs and tissues through the action of specific oxidases [*e.g.*, NADPH oxidase (NOX2)], peroxidases (*e.g.*, myeloperoxidase), and as a by-products of mitochondrial electron transport chain (Altenhofer et al., 2012). RNS are a family of molecules derived from nitric oxide (•NO) produced by neuronal, endothelial, and inducible isoforms of nitric oxide synthase (NOS). Phagosomal activation of NOX2 and iNOS serves as primary source of O_2_•− and •NO that react together to form highly stable and cytotoxic peroxynitrite (ONOO−) required for direct killing of *T. cruzi* in macrophages (Alvarez et al., 2011). While ROS/RNS play an important role in signaling host defense and regulation of pH and ion concentration in the phagosome and in cell differentiation (Bedard and Krause, 2007), excessive oxidative/nitrosative stress can cause a wide range of pathological processes and cell and tissue injury in the host. In *T. cruzi* infection, NOX2 components were detected at the plasma membrane of peritoneal macrophages (de Carvalho and de Souza, 1987). However, splenocytes of infected mice and macrophages *in vitro* infected with *T. cruzi* exhibited low levels of ROS and •NO production that allowed parasite survival (Koo et al., 2016). Myeloperoxidase and nitrite levels were increased in circulation of *T. cruzi*-infected mice (Dhiman et al., 2008) and humans (Dhiman et al., 2009), though their relevance in parasite control is not clear.

Mitochondrial respiratory chain is the site for leakage of electrons to oxygen and superoxide production. In context of CD, we have documented that infection by *T. cruzi* results in intracellular Ca^2+^ flux, changes in mitochondrial membrane potential, and a decline in the activities of the respiratory complexes. Consequently increase in electrons’ leakage to O_2_ and increased O_2_•− formation were noted in murine cardiomyocytes (Gupta et al., 2009; Wen and Garg, 2010) and almost all muscle tissues of acutely infected mice (Gupta et al., 2009). Mitochondrial defects of complex III and resultant increase in ROS release persisted in the heart tissue of chronically infected mice and Chagas patients (Wen et al., 2008; Wen and Garg, 2010; Wan et al., 2012; Dhiman et al., 2013), thus suggesting that mitochondria are a major source of oxidative stress after acute infection phase and during chronic phase of disease progression. ROS-induced oxidative adducts were conducive to proinflammatory macrophage activation (Choudhuri and Garg, 2020).

**Figure 3:**
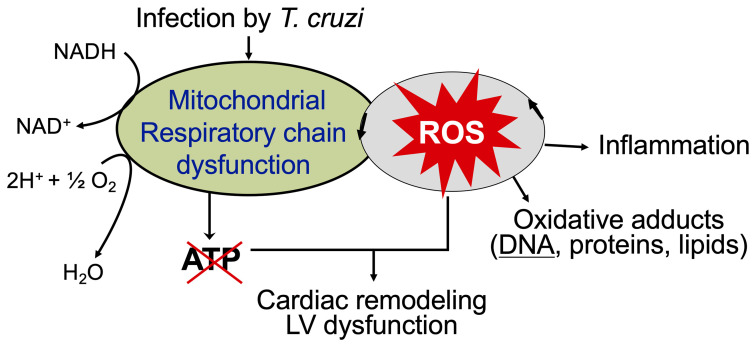
Mitochondrial ROS contribute to cardiac damage, inflammation, and remodeling in chronic Chagas cardiomyopathy. Mitochondrial dysfunction of electron transport chain is sustained in chronically infected mice and humans that results in persistence of ROS production and a decline in oxidative phosphorylation and ATP production. ROS-induced oxidative adducts signal inflammatory responses. Antioxidants capable of scavenging ROS have a potential to control cardiac damage and remodeling in chronic CD.

The overall cellular oxidative stress is an outcome of the relative rate of ROS production and the rate by which ROS are reduced by antioxidants. The critical antioxidants in cardiomyocytes including Mn^2+^ superoxide dismutase (MnSOD), glutathione peroxidase, catalase, and glutathione were enhanced in various muscle tissues of acutely infected mice (Wen et al., 2004). However, progression of chronic phase in infected mice and humans was associated with sustained or increase in mitochondrial ROS release and oxidative stress markers (*e.g.*, glutathione disulfide, lipid peroxide, protein carbonyl, 4-hydroxynonenal) as well as non-responsive or decreased antioxidants (especially MnSOD) activity (Perez-Fuentes et al., 2003; Budni et al., 2013; Dhiman et al., 2013). Moreover, treatment with an antioxidant controlled the oxidative insult in infected patients (Ribeiro et al., 2010) and preserved the mitochondrial and cardiac function in infected mice (Wen et al., 2006; Wen et al., 2010). Phenyl-N-tert-butylnitrone (PBN) is a spin-trapping antioxidant. Treatment of *T. cruzi*-infected mice and rats with PBN was beneficial in arresting the myocardial oxidative lesions and preserving the rate of oxidative phosphorylation and ATP synthesis (Wen et al., 2006; Wen et al., 2010). Likewise, MnSOD transgenic mice were better equipped in controlling the inflammatory infiltrate and oxidative stress and cardiac hypertrophy that are hallmarks of chronic CD (Dhiman et al., 2013). All these observations suggest that inefficient antioxidant response, along with mitochondrial dysfunction, contributes to sustained oxidative and inflammatory stress in Chagas cardiomyopathy (**Figure 3**).

## SIRT1: A Potential Adjuvant Therapy in CD

### SIRT1 Agonists

Resveratrol and SRT1720 are the most studied SIRT1 agonists in scientific literature. Resveratrol (3,5,4′-trihydroxy-*trans*-stilbene) is a natural phytoallexin found in grapes’ skin, berries, peanuts, and roots of rhubarb, and other plants. Resveratrol protects the plants from fungal infection, and it was first described in 1940s. In 2003, a drug screen for small molecule activators of SIRT1 identified fifteen SIRT1 activators, and resveratrol was the most potent one. The anti-aging properties of resveratrol were evidenced by an increase in the life span of yeast, nematodes, fish, and flies *etc. (* Valenzano et al., 2006; Agarwal and Baur, 2011). Others documented that resveratrol is broadly beneficial in inhibiting the growth of cancer cells in culture (Saunier et al., 2017), and in preventing or slowing down the neurodegenerative disorders, cardiac involvement, and cancer in rodents (Hung et al., 2000; Ignatowicz and Baer-Dubowska, 2001; Shigematsu et al., 2003). Moreover, resveratrol treatment of mice improved the running time and increased oxygen consumption in the skeletal muscle, which was associated with activation of genes for oxidative phosphorylation and mitochondrial biogenesis (Guo et al., 2014). The authors attributed these benefits to activation of SIRT1 and PGC-1*α* and found that resveratrol treatment prevented the diet-induced-obesity and insulin resistance in mice. Cumulatively, many studies have shown that resveratrol offers a multitude of benefits including increase in mitochondrial content and antioxidant capacity, reduced inflammation, improved metabolic and vascular function (Thirunavukkarasu et al., 2007; Barger et al., 2008; Bereswill et al., 2010; Guo et al., 2014).

SRT1720 was first identified in a high-throughput *in vitro* fluorescence polarization assay which was used for screening SIRT1 agonists (Milne et al., 2007). SRT1720 is ~1,000-fold more potent than resveratrol in activating SIRT1 activity. SRT1720 exerts its effects by binding to SIRT1–substrate complex and potentiate deacetylation of SIRT1 target proteins (Milne et al., 2007; Feige et al., 2008). C2C12 cells treated with SRT1720 exhibited increased expression of citrate synthase activity and ATP levels indicating the improvement of mitochondrial biogenesis (Smith et al., 2009). *In vivo* studies in obese mice and rats showed that SRT1720 treatment improved the insulin sensitivity, regulated plasma glucose levels, and enhanced the mitochondrial oxidative metabolism in various tissues, including skeletal muscle, liver, and brown adipose tissue (Minor et al., 2011; Durkacz et al., 1980; D’Amours et al., 1999). We demonstrated that SIRT1 activity was decreased in mice infected with *T. cruzi*, and treatment with SRT1720 during the indeterminate phase, *i.e.*, after the immune control of circulating, blood parasitemia and before the onset of chronic disease phase, preserved the cardiac structure and function in CD mice (Wan et al., 2016).

Some studies have noted that resveratrol and SRT1720 exhibit off-target activities against many enzymes, ion channels, receptors, and transporters and do not directly influence SIRT1-mediated deacetylation on target peptides (Pacholec et al., 2010). Nevertheless, because of its small molecular size and potent activity, SRT1720 is actively being studied for the treatment of metabolic and chronic diseases.

### SIRT1 Improves Mitochondrial Biogenesis

One of the primary targets of SIRT1 deacetylation is PGC-1*α*. PGC-1*α* undergoes post-translational modifications including acetylation and phosphorylation, and it serves as master regulator of the mitochondrial biogenesis and oxidative metabolism. Studies have shown that deacetylation of PGC-1*α* is dependent on SIRT1 activity (Sims et al., 1981; Gerhart-Hines et al., 2007; Amat et al., 2009; Gurd, 2011; Guo et al., 2014). Mutation of the acetylation sites, which sealed PGC-1*α* in deacetylated state, markedly enhanced its basal activity (Sims et al., 1981). It was suggested that SIRT1 may physically and functionally interact with PGC-1*α* through ADP-ribosyl transferase domain of SIRT1, and mutations in this domain prevent SIRT1’s interactions with PGC-1*α* (Gurd, 2011). SIRT1-activated PGC-1*α* rapidly translocates to the nucleus where it co-activates the NRF1/NRF2 and TFAM to regulate the expression of genes encoding key components of respiration, mtDNA transcription, and mtDNA replication machineries. SIRT1 also acts as a sensor of changes in nutrient and energy metabolism. SIRT1 was activated by fasting, and SIRT1 and PGC-1*α* interaction enhanced the expression of hepatic gluconeogenic genes (Sims et al., 1981). SIRT1 deacetylation of PGC-1*α* in skeletal muscle was required for signaling the expression of enzymes involved in mitochondrial fatty acid oxidation (Gerhart-Hines et al., 2007).

Poly(ADP-ribose) polymerase (PARP) family members are recognized for their function in maintaining DNA and RNA metabolism. Among the 17 known members of the PARP family, PARP1 is most active enzymatically. PARP1 is activated upon sensing the oxidized DNA (Beck et al., 2014), and it utilizes NAD as a substrate to catalyze the formation of poly(ADP-ribose) (PAR) chains on itself and other proteins (D’Amours et al., 1999; Hassa et al., 2006; Messner et al., 2010). SIRT1 and PARP1 compete for NAD^+^ availability; crosstalk between these proteins is inevitable (Luna et al., 2013). PARP1 knockout mice exhibited an increase in mitochondrial content and energy expenditure and were protected against metabolic disease (Hassa et al., 2006) confirming that PARP1 influences SIRT1 activity in maintaining mitochondrial homeostasis. In mice chronically infected with *T. cruzi*, myocardial PARP1 expression was increased, and it compromised the polymerase gamma activity resulting in a decline in mtDNA content, mtDNA-encoded gene expression, and oxidative phosphorylation capacity (Wan et al., 2016; Wen et al., 2018). Consequently, an increase in mitochondrial ROS and oxidative stress was noted in Chagas myocardium. Genetic deletion of PARP1 or treatment with PARP1 inhibitor or SIRT1 agonist improved the mitochondrial biogenesis, oxidative phosphorylation, and left ventricular function in chronic CD (Wan et al., 2016; Wen et al., 2018). These studies indicate the SIRT1–PARP1 imbalance is a major contributor to myocardial mitochondrial stress in CD.

### SIRT1 Mitigates Oxidative Stress

Oxidative stress is characterized by increased intracellular levels of ROS and decreased antioxidant capacity (Johnson and Giulivi, 2005). Oxidative stress plays a key role in the pathophysiology of many diseases, including atherosclerosis, diabetic mellitus, and myocardial dysfunction of infectious and non-infectious etiologies (Deng et al., 2008).

SIRT1 has been demonstrated to control ROS levels by its regulatory effects on mitochondrial electron transport chain (discussed above). SIRT1 also inhibited NF-*κ*B transcriptional activation and reduced the expression of gp91^phox^ and p22^phox^ encoding for NADPH oxidase subunits, and thus prevented the production of reactive oxygen and nitrogen radicals by phagocytes (Xie et al., 1994; Sakitani et al., 1998; Anrather et al., 2006; Manea et al., 2007; Manea et al., 2010). Quercetin enhanced the SIRT1 expression, increased AMP-activated protein kinase (AMPK) activity, and decreased NADPH production, thus providing protective effects against the hyperglycemia-induced oxidant damage in HUVEC cells (Hung et al., 2015). Conversely, SIRT1 inhibition led to increase in NOX2 activity and ROS production and contributed to endothelial dysfunction (Wosniak et al., 2009; Zarzuelo et al., 2013).

At a molecular level, SIRT1 influences antioxidant status through its effects on transcription factors. FoXO (Forkhead box) family of transcription factors consists of FoxO1, FoxO3, FoxO4, and FoxO6 (Kaestner et al., 2000). FoXO proteins regulate many cellular processes, including stress resistance, energy metabolism, cell cycle, and cell death. In a number of studies, SIRT1 is shown to deacetylate FoxO1, FoxO3a, and FoxO4, and activate FoXO-regulated antioxidants in mitochondria (*e.g.*, MnSOD, peroxiredoxins 3 and 5), peroxisomes (catalase), and plasma (e.g., selenoprotein P, ceruloplasmin) (Zhang and Tarleton, 1999; Brunet et al., 2004; van der Horst et al., 2004; Sengupta et al., 2011; Xiong et al., 2011; Yamamoto and Sadoshima, 2011). SIRT1-mediated deacetylation of FoXO1 and FoXO4 enhanced their DNA-binding ability to induce antioxidants’ gene expression (Daitoku et al., 2004; van der Horst et al., 2004). FoxO1 depletion by siRNA inhibited SIRT1 expression in vascular smooth muscle cells and HEK293 cells suggesting that FoxO1 provides positive feedback signal for SIRT1 activity (Xiong et al., 2011).

Nuclear Factor, Erythroid 2 Like 2 (NFE2L2, also referred as NRF2 in literature) bind to the antioxidant response element (ARE) sequences in promoters of the antioxidant genes and plays an important role in activation of cellular antioxidant defense. It has been reported that melatonin signals SIRT1-dependent transcriptional activation of NRF2 to exert anti-oxidative effects in the developing rat brain and BV2 cells, and SIRT1 inhibitor significantly decreased the SIRT1 and NRF2 expression in BV2 cells (Shah et al., 2017). SIRT1 was also demonstrated to enhance the activation of the NFE2L2 (NRF2)/ARE antioxidant pathway and inhibit the apoptosis of type II alveolar epithelial cells (Ding et al., 2016). We noted that *T. cruzi* induced ROS had a negative effect on the expression, nuclear translocation as well as ARE binding and transcriptional activity of NFE2L2 in cardiomyocytes (Wen et al., 2017). Subsequently, expression of several of the NFE2L2-regulated antioxidants, including gamma-glutamyl cysteine synthase, heme oxygenase-1, glutamate-cysteine ligase, thioredoxin, glutathione S transferase, and NADPH dehydrogenase quinone 1 was decreased in cardiomyocytes and myocardium of mice infected with *T. cruzi (* Wen et al., 2017 *)*. Though we did not examine the effects of SIRT1 agonist on NFE2L2 in this study; however, preserving the NFE2L2 activity by other means established the antioxidant/oxidant balance and preserved the mitochondrial and cardiac health in CD mice (Wen et al., 2017).

### SIRT1 Controls Inflammation

The NF-*κ*B transcription factor family consists of five proteins including p65 (RelA), RelB, c-Rel, p105/p50 (NF-*κ*B1), and p100/52 (NF-*κ*B2). NF-*κ*B transcription factors regulate the expression of a large number of genes involved in inflammation, cell proliferation, cell differentiation, and cell survival. In the absence of stimuli, NF-*κ*B remains in an inactive form in the cytoplasm through association with inhibitory I*κ*B proteins. I*κ*B kinase (IKK)-mediated I*κ*B phosphorylation leads to its degradation and released NF-*κ*B translocates to nucleus to carry out transcription of target genes (Nakajima and Kitamura, 2013). SIRT1 deacetylates the p65/RelA at Lys^310^ residue and inhibits NF-*κ*B activity (Yeung et al., 2004). SIRT1 can also inhibit NF-*κ*B by activating AMPK and peroxisome proliferator-activated receptor *α*, which inactivate the NF-*κ*B pathway and prevent ROS generation. Resveratrol treatment enhanced the SIRT1 activity, and repressed NF-*κ*B and ROS generation in HUVEC cells treated with TNF-α (Pan et al., 2016). NF-*κ*B can also suppress SIRT1 activity (Kauppinen et al., 2013), thus suggesting that a feedback regulation of SIRT1 and NF-*κ*B determines the inflammatory and oxidative status in a cell.

Acute inflammation occurs over seconds to days to eliminate the cause of tissue injury (*e.g.*, microbes) and reinstates the immune homeostasis. Innate immune cells including monocytes/macrophages, granulocytes, and dendritic cells express toll-like receptors (TLRs), nod like receptors (NLRs), rig-I-like receptors (RLRs), and other receptors that sense pathogen-associated molecular patterns (PAMPs) and signal transcription activation of cytokines and chemokines (Dalpke and Heeg, 2002). Further, TLR sensing of PAMPs rapidly stimulates the expression and transcriptional activation of Hypoxia Inducible Factor 1 Subunit Alpha (HIF-1*α*), which switches mitochondrial glucose oxidation to glycolysis and increase in glucose level for supporting the expression of proinflammatory genes (Koo and Garg, 2019). Transcription regulation of the switching from the proinflammatory to anti-inflammatory and immuno-regulatory state requires SIRT1-dependent deacetylation of NF-*κ*B and histone proteins (*e.g.*, H1K26, H4K16, H3K9, and H3K14) and recruitment of new methyltransferases (Liu and McCall, 2013). SIRT1 also regulates acute inflammatory process by mediating a metabolic switch (Krawczyk et al., 2010; Michalek et al., 2011; Liu et al., 2012) through activating PGC-1*α* that increases the flux of fatty acids and transfer of fatty acids into mitochondria and supports fatty acid oxidation as an energy source for mitochondrial function (Liu and McCall, 2013).

Chronic inflammation is caused by persistent low-level of pathogenic or non-pathogenic stimuli. A decline in SIRT1 activity is noted in many chronic inflammatory diseases. Examples include fat deposits in obesity (Schug and Li, 2010), lungs in chronic obstructive pulmonary disease (COPD) (Rajendrasozhan et al., 2008), brain in Alzheimer’s disease (Qin et al., 2006), arteries in atherosclerosis (Gorenne et al., 2013), and skin in aging (Massudi et al., 2012). SIRT1 inhibition likely contributes to chronic inflammation due to hosts’ inability to shut down NF-*κ*B-dependent gene expression of proinflammatory cytokines (Serrano-Marco et al., 2012). Yet, the mechanisms of SIRT1 decline during chronic inflammation are not very clear. Enhancing NAD^+^ levels (Imai and Guarente, 2014) or activating SIRT1 by resveratrol suppressed chronic inflammation and helped establish metabolic homeostasis (Haigis and Sinclair, 2010). It is suggested that cellular bioenergetics and SIRT1 coordinate to provide a regulation axis (positive and negative) that controls host’s inflammatory response to pathogens as well as maintain cellular metabolic homeostasis (Liu et al., 2011).

We showed that yolk-sac-derived CD11b^+^ F4/80^+^ monocytes/macrophages were increased in spleen and heart tissue of CD mice, and these cells displayed surface markers of inflammatory phenotype (CD80^+^/CD64^+^ > CD200^+^/CD206^+^) as well as inflammatory functional response, evidenced by increase in the cytokines’ expression (IL-6 + TNF-α >> Arg-1 + IL-10) (Wan et al., 2019). When treated with SRT1720, splenic expansion and myocardial infiltration of proinflammatory monocytes/macrophages were controlled in CD mice. SRT1720 did not alter the inherent capability of macrophages to respond to *T. cruzi* infection. Instead, SRT1720 treatment diminished the *T. cruzi*-induced expression and/or phosphorylation of focal adhesion kinase (FAK) non-receptor kinase and the downstream transcription factors (Pu.1, c-Myb, and Runx1) that are known to participate in macrophage proliferation and migration and Notch1 that is involved in macrophage’s functional activation. Studies in cultured macrophages showed that SIRT1 agonist or FAK inhibitor abrogated the NF-*κ*B transcriptional activity and inflammatory cytokine gene expression in macrophages infected with *T. cruzi*, and thus provided further evidence of agonistic effects of SIRT1 on FAK signaling of transcription factors involved in macrophage functional activation (Wan et al., 2019).

## Conclusive Remarks

**Figure 4:**
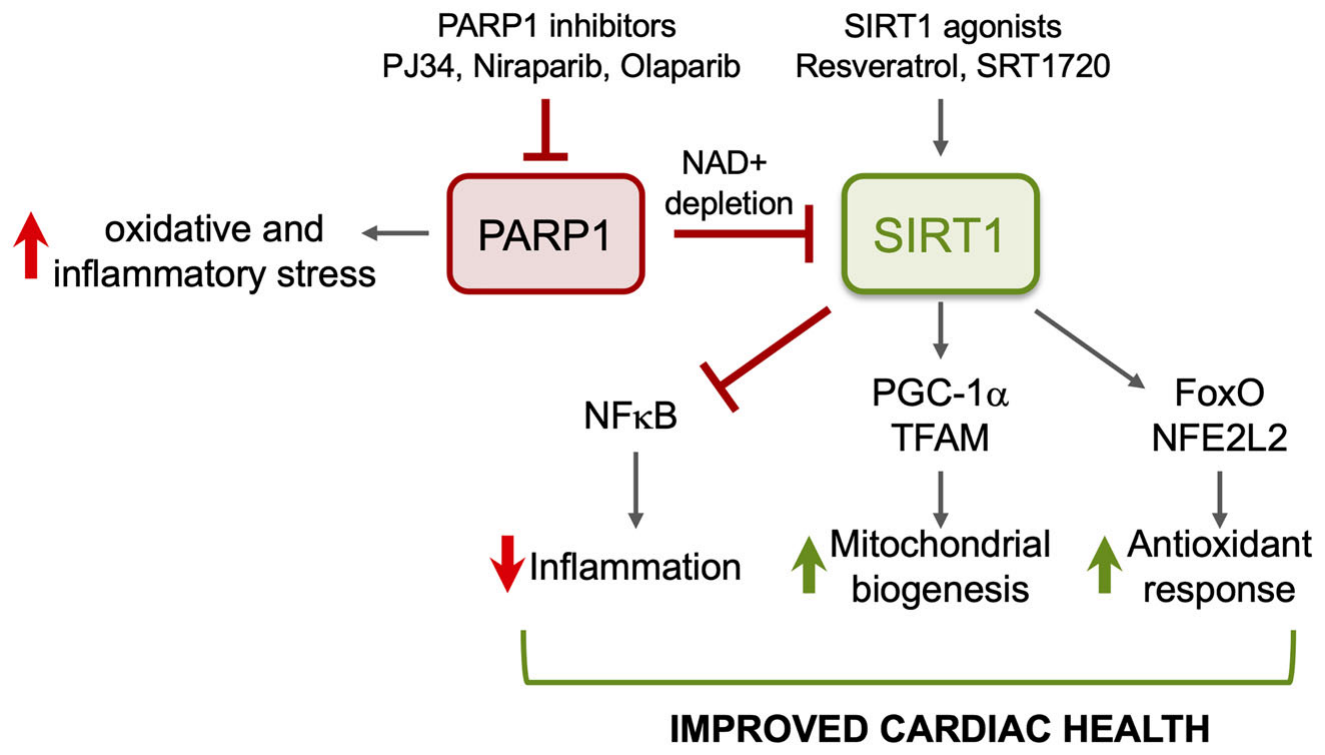
Potential benefits of SIRT1 agonists in CD. ROS induced oxidative adducts cause PARP1 hyperactivation that suppresses mitochondrial DNA replication and transcription and signals inflammation. PARP1 suppresses SIRT1 activity in Chagasic heart through depletion of NAD^+^ pool. Pharmacological inhibitors of PARP1 or activators of SIRT1 will enhance SIRT1-mediated deacetylation and will have cytoprotective effects in Chagas disease through inhibition of oxidative stress and inflammation and induction of antioxidant response and mitochondrial biogenesis.

Inflammatory and oxidative stress and mitochondrial dysfunction are recognized of pathological importance in chronic Chagas cardiomyopathy. We have shown that SIRT1 activity is compromised in the myocardium during CD progression, and treatment of infected mice with SRT1720 for 3-weeks in indeterminate phase ameliorated the left ventricular dysfunction. The benefits of SRT1720 were associated with control of chronic oxidative stress and proinflammatory differentiation of macrophages in spleen of chronically infected mice (**Figure 4**). Similar benefits in improving the cardiac outcomes in infected mice were obtained by inhibiting PARP1 that competes with SIRT1 for the NAD^+^ substrate. Whether PARP1–SIRT1 imbalance occurs in human Chagas disease and whether PARP1 inhibitors or SIRT1 agonists will be useful in improving the cardiac outcomes in human Chagas disease remain to be investigated in future studies.

The mechanism(s) connecting SIRT1 to tissue inflammation/oxidative stress have been enigmatic. The bone marrow and splenic myeloid progenitor cells give rise to proinflammatory macrophages in Chagas heart. However, how SIRT1 deficiency and SIRT1 agonists regulate the proliferation and differentiation of monocytes of different lineages into macrophages with diverse phenotypic and functional activation remains to be studied. Likewise, how the crosstalk or substrate allocation between SIRT1 and PARP1 is regulated is not known, and further studies will be needed to understand their precise role in human health and disease, especially as related to Chagas disease.

## Author Contributions

XW wrote the first draft. XW and NG edited and formatted the later drafts. All authors contributed to the article and approved the submitted version.

## Funding

NJG has been supported by grants from the National Institute of Allergy and Infectious Diseases (R01AI136031; R01AI054578) and National Heart Lung and Blood Institute (R01HL094802) of the National Institutes of Health. XW was supported by American Heart Association pre-doctoral fellowship and Kempner post-doctoral fellowship. The funders had no role in study design, data collection and analysis, decision to publish, or preparation of the manuscript.

## Conflict of Interest

The authors declare that the research was conducted in the absence of any commercial or financial relationships that could be construed as a potential conflict of interest.

## Abbreviations

CD, Chagas disease; mtDNA, mitochondrial DNA; PGC-1*α*, peroxisome proliferator–activated receptor gamma coactivator 1; RNS, reactive nitrogen species; ROS, reactive oxygen species; SIRT1, sirtuin 1.
